# Reference Values of M-mode Echocardiographic Parameter in Adult Toy Breed Dogs

**DOI:** 10.3389/fvets.2022.918457

**Published:** 2022-06-23

**Authors:** Noriko Isayama, Yusuke Uchimura, Kenta Sasaki, Erika Maeda, Toshihisa Takahashi, Megumi Watanabe

**Affiliations:** Department of Cardiology, Ueno no Mori Animal Hospital, Tokyo, Japan

**Keywords:** M-mode echocardiography, myxomatous mitral valve disease, mitral regurgitation, LVIDDN, toy breed dogs

## Abstract

**Introduction:**

Myxomatous mitral valve disease (MMVD) is one of the most common heart diseases in dogs, and there is a dearth of reports that have investigated reference values for left ventricular end-diastolic internal diameter corrected for body weight (LVIDDN) exclusively in toy breeds.

**Animals:**

Eighty-six client-owned healthy dogs weighing <5 kg, including Toy Poodles, Chihuahuas, Yorkshire Terriers, Papillon, and other small breeds or small mixed breeds (mixed breed, Pomeranian, dachshund, Shih Tzu, and Maltese). In this retrospective single-center study, data were collected from dogs attending clinic for annual checkup between April 2014 and March 2021.

**Materials and Methods:**

Experienced echocardiographers performed transthoracic echocardiography, with reference ranges established using healthy dogs. Measurements of body weight (BW), heart rate, and several echocardiographic variables were obtained. The association between BW and echocardiographic parameters was assessed by linear regression analyses. M-mode measurements were obtained and normalized using equations developed from the regression analyses.

**Results:**

The LVIDDN value for 95% of dogs weighing <5 kg was achieved by dividing the M-mode measurement by BW raised to the power 0.332. The upper limit of the prediction interval for breeds weighing <5 kg was much lower than the value currently applied.

**Conclusions:**

We propose a reference LVIDDN value of ≥1.6 for the diagnosis of stage B2 MMVD in toy breed dogs. The results of our study will guide clinicians in deciding when to start treatment for MMVD in small breed dogs.

## Introduction

Myxomatous mitral valve disease (MMVD) is one of the most common heart diseases in dogs. In North America, an estimated 75% of heart disease cases in dogs are attributed to MMVD (1–3). A polygenic mode of inheritance of the disease is evident, along with an observed breed specificity, as MMVD is predominantly present in relatively smaller breeds with an average adult weighing <9 kg (4, 5). The prevalence of MMVD has highlighted the need for regular monitoring, as noted in the American College of Veterinary Internal Medicine (ACVIM) consensus statements, of smaller breed dogs for the presence of MMVD (3, 6). To evaluate cardiac anatomy and function in the context of small animal cardiology, echocardiography is considered the most useful diagnostic tool (7). To measure the severity of cardiac enlargement, measurement of left ventricular end-diastolic internal diameter corrected for body weight (BW) (LVIDDN)—a parameter important for the treatment of MMVD according to the ACVIM consensus statement- is required (8). One of the four criteria that identifies advanced stage B2 of MMVD in dogs is an increased value of LVIDDN to ≥1.7 (3).

Several veterinary studies have suggested that body size or BW imparts clinically relevant impacts on echocardiographic variables in animals (9–13). However, the existing LVIDDN reference values do not include toy breeds in the data, and a wide range of weight of dogs is included in calculating the current reference values. For example, in one of the seminal studies that evaluated a large sample of dogs of different breeds and a wide variety of body sizes to predict normal M-mode measurements in adult dogs, the number of small breed dogs (<5 kg) was relatively small and did not include toy breeds, such as Chihuahua, Toy Poodle, the breeds commonly seen in Japan, and this fact possibly masked the appropriate threshold levels for these smaller breeds (8). Although, breed specific M-mode measurement data for miniature poodle and Dachshund are reported in literature following logarithmic analysis of previous studies, there is a dearth of reports that have investigated the LVIDDN reference values exclusively for toy breeds (14).

As a lack of standardized LVIDDN values can impact the clinical practice to treat stage B2 MMVD, it is imperative to revisit the calculation and check if the standardized value of LVIDDN holds true for small dogs. Therefore, this study aimed to estimate the LVIDDN reference values of small breed dogs weighing <5 kg. To the best of our knowledge, this is one of the first studies to assess the reference range for LVIDDN values in adult toy breed dogs.

## Animal, Materials and Methods

This retrospective review of clinical records was conducted between April 2014 and March 2021 in our clinic. All procedures in this study were approved by the owners, and verbal or written informed consent to conduct and publish the study was obtained from the owners. The study protocol was reviewed and approved by the Institutional Review Board of Ueno no Mori Animal Hospital (approval number 1404-01).

This study consisted of client-owned clinically adult dogs of different breeds, including Toy Poodles, Chihuahuas, Yorkshire Terriers, Papillon, and other small breeds or small mixed breeds (mixed breed, Pomeranian, Dachshund, Shih Tzu, and Maltese). The dogs were of both sexes (M/F), spayed female (S), and castrated male (C), and were examined by a single investigator using the same protocol and instruments.

Data were collected retrospectively from the clinical records of 680 dogs who visited the clinic for an annual checkup during the study period. Healthy dogs weighing <5 kg and free of respiratory or cardiac disease based on echocardiography, electrocardiography, blood pressure tests, and blood tests were included in the study. Dogs with body condition score 3–7 were included. Dogs with extreme obesity and emaciation were considered unhealthy and excluded. Other exclusion criteria included gallop rhythm, pathological heart murmur, or nonsinus arrhythmia, presence of any systemic illness based on history and physical examination, cardiac abnormalities identified in a baseline M-mode, 2D, and Doppler echocardiography, and uncooperative temperament for echocardiography. The dogs were evaluated in the right and left lateral recumbency while being restrained manually without any sedation.

### Conventional Echocardiography

Experienced echocardiographers performed transthoracic echocardiography using an ultrasonographic unit with a 2.4-8.0-MHz probe. To establish reference ranges, a single investigator obtained the echocardiograms of healthy dogs. Raw imaging data from each study were captured digitally for offline analysis, which was later performed using a digital workstation.

BW, heart rate, and the following echocardiographic measurements were made: left ventricular end-diastolic internal diameter (LVIDd), left ventricular end-systolic internal diameter (LVIDs), and interventricular septal thickness in diastole (IVSd), interventricular septal thickness in systole (IVSs) and left ventricular free wall thickness in systole (LVWs), and left ventricular posterior wall end-diastole (LVPWd) of 86 dogs were collected as previously described (15).

The short-axis M-mode LVIDd measurement was indexed to BW, following a previous study (8). Fractional shortening (Fs) was calculated as [LVIDd]–[LVIDs]) / LVIDd ×100.

In addition, left atrial (LA) and aortic root dimensions at end-diastole (Ao) diameter were measured in the right parasternal in B-mode 2D using a short-axis view at the aortic valve level as described previously (16). In addition to the measured values, the ratio of left atrial to aortic root diameter (LA/Ao) was calculated. Using pulsed-wave Doppler echocardiography in the left parasternal apical four-chamber view, transmitral flow velocity was measured with the sample volume positioned at the tip of the mitral valve leaflets. The mitral early diastolic flow (E wave) and late diastolic flow (A wave) velocities were measured, and the ratio of the E to A wave (E/A ratio) was calculated.

### Data Analysis

All statistical analyses were performed with R version 3.6.2 (R Core Team 2019, R Foundation for Statistical Computing, Vienna, Austria). Assuming that a non-linear relationship exists (allometric scale rule) between BW and echocardiographic parameters (LVIDd and LVIDDN), The association between BW and echocardiographic parameters was assessed by linear regression analyses. We evaluated the relationship between LVIDd and BW based on previous studies and the following relationship equation (linear regression model) (8).


$$\begin{array}{l} \text{Log (LVIDd)}=\log\text{(a) + b}\times \log\;\left(\text{weight}\right). \end{array}$$

**Figure 1:**
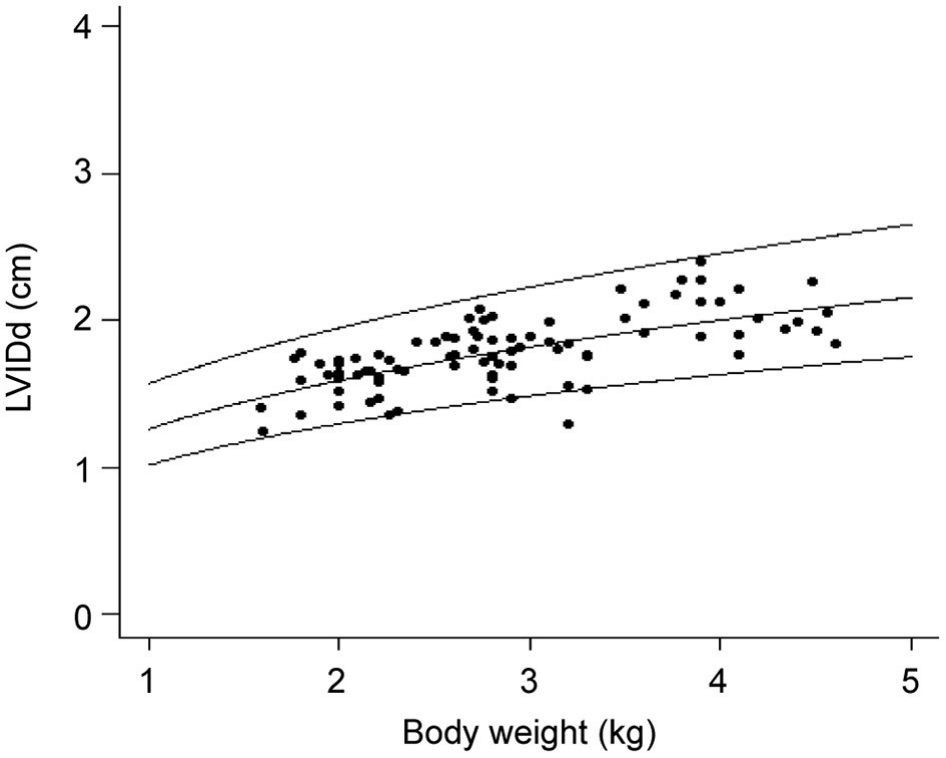
Scatter plot of LVIDd and body weight with predicted values and 95% confidence intervals. LVIDd, Left ventricular end diastolic internal diameter.

The 95% prediction intervals (PIs) were calculated and drawn on a scatter plot with weight as the x-axis and echocardiographic parameters as the y-axis to visually evaluate the association (Figure 1). The slope of the regression line gives the constant b in the allometric equation, and the antilogarithm (log-1) of the intercept of the line gives the constant a (8).

M-mode measurements could be normalized by using the equations developed from the regression analyses. The allometric equation was rearranged to achieve the proportionality constant, a = Y/Mb. If Y is an M-mode measurement and Mb is the BW raised to the power b, a is a constant, that is, the normalized or indexed M-mode measurement. For dogs of varying weights, the normal M-mode average values and PIs are presented in Table 1. The LVIDDN presented in Table 1 was calculated using the scaling exponent b = 0.294.

**Table 1:** Calculation of basic statistics of dogs used in the study.

	**Toy Poodle**		**Chihuahua**		**Yorkshire**		**Papillon**		ALL*(including 10 dogs of	
	**(*****N*** **= 40)**		**(*****N*** **= 25)**		**Terrier (*****N*** **= 7)**		**(*****N*** **= 4)**		**other small breed categories) (*****N*** **= 86)**	
	**Mean**	**Median**	**Mean**	**Median**	**Mean**	**Median**	**Mean**	**Median**	**Mean**	**Median**
	**(standard**	**(25–75%)**	**(standard**	**(25–75%)**	**(standard**	**(25–75%)**	**(standard**	**(25–75%)**	**(standard**	**(25–75%)**
	**deviation)**		**deviation)**		**deviation)**		**deviation)**		**deviation)**	
BW	2.903 (0.8450)	2.800 (2.125–3.825)	2.601 (0.7430)	2.340 (2.200–2.800)	2.651 (0.3225)	2.700 (2.620–2.810)	3.635 (0.4908)	3.450 (3.300–3.785)	2.869 (0.7985)	2.780 (2.200–3.300)
Age	5.700 (3.2439)	5.000 (3.000–8.250)	6.840 (3.7714)	7.000 (3.000–10.000)	5.857 (2.5448)	6.000 (4.500–6.000)	9.000 (1.8257)	9.000 (7.750–10.250)	6.186 (3.5097)	6.000 (3.000–9.000)
HR	132.421 (25.1145)	136.000 (116.000–148.500)	123.708 (19.7473)	123.000 (114.500–133.250)	131.000 (25.3509)	129.000 (117.500–151.500)	136.750 (23.7680)	133.000 (118.750–151.000)	130.036 (23.3078)	130.000 (115.500–145.000)
IVSd (cm)	0.607 (0.0982)	0.615 (0.550–0.670)	0.555 (0.1803)	0.550 (0.520–0.650)	0.542 (0.0873)	0.510 (0.469– 0.610)	0.712 (0.0699)	0.730 (0.679–0.763)	0.591 (0.1286)	0.590 (0.530–0.670)
LVIDd (cm)	1.824 (0.2486)	1.758 (1.649–2.002)	1.692 (0.1988)	1.720 (1.583–1.853)	1.876 (0.1134)	1.896 (1.881–1.912)	1.840 (0.2513)	1.855 (1.710–1.985)	1.788 (0.2422)	1.770 (1.633–1.924)
LVPWd (cm)	0.576 (0.0836)	0.570 (0.500–0.640)	0.568 (0.0782)	0.570 (0.510–0.610)	0.535 (0.0545)	0.534 (0.495–0.550)	0.718 (0.0754)	0.745 (0.692–0.770)	0.579 (0.0850)	0.570 (0.515–0.640)
LVIDDN**	1.342 (0.1164)	1.324 (1.265–1.416)	1.288 (0.1317)	1.307 (1.175–1.382)	1.410 (0.0549)	1.413 (1.373–1.434)	1.259 (0.1545)	1.253 (1.204–1.309)	1.320 (0.1307)	1.321 (1.247–1.414)
Fs	45.477 (5.4663)	46.150 (41.125–48.000)	40.153 (6.6893)	40.000 (36.200–43.250)	45.649 (3.9851)	47.900 (42.050–48.200)	40.005 (7.4515)	40.050 (36.830–43.225)	42.907 (6.4763)	42.800 (38.850–47.525)
IVSS	0.824 (0.1271)	0.860 (0.723–0.930)	0.769 (0.1117)	0.740 (0.695–0.822)	0.825 (0.1277)	0.875 (0.790–0.910)	0.845 (0.0705)	0.840 (0.788–0.897)	0.801 (0.1197)	0.780 (0.720–0.900)
LVIDS	1.032 (0.2161)	1.000 (0.899–1.117)	1.017 (0.2190)	0.995 (0.901–1.152)	1.010 (0.1160)	1.003 (0.947–1.113)	1.087 (0.0352)	1.075 (1.067–1.095)	1.043 (0.2097)	1.030 (0.909–1.130)
LVWS	0.805 (0.1249)	0.810 (0.730–0.887)	0.751 (0.1056)	0.745 (0.660–0.812)	0.790 (0.0935)	0.795 (0.732–0.853)	0.932 (0.0299)	0.940 (0.928–0.945)	0.785 (0.1214)	0.770 (0.690–0.885)
AO (cm)	1.720 (2.6952)	1.095 (0.990–1.240)	1.071 (0.1309)	1.090 (0.950–1.162)	2.214 (2.9488)	1.060 (1.000–1.320)	1.193 (0.0208)	1.200 (1.185–1.205)	1.489 (2.0390)	1.100 (1.000–1.210)
LA (cm)	2.226 (3.3437)	1.275 (1.150–1.415)	1.306 (0.2285)	1.305 (1.118–1.452)	2.741 (3.7058)	1.400 (1.195–1.615)	1.693 (0.0231)	1.680 (1.680–1.700)	1.895 (2.5430)	1.310 (1.150–1.560)
E. (cm/s)	59.386 (23.7110)	65.500 (55.800–73.100)	60.572 (17.6177)	64.400 (52.400–71.500)	65.400 (9.0620)	61.200 (60.200–68.500)	50.000 (4.8497)	50.800 (47.800–52.600)	57.475 (22.5066)	62.800 (51.250–71.550)
A. (cm/s)	57.877 (25.8087)	60.300 (45.100–75.200)	58.523 (17.2812)	59.000 (55.000–66.200)	64.633 (10.4433)	67.700 (60.350–70.450)	65.933 (7.8002)	66.000 (62.050–69.850)	56.824 (23.4261)	59.000 (50.750–71.400)
DcTms	92.745 (29.1268)	83.850 (74.375–101.900)	70.327 (18.7299)	68.000 (61.400–76.150)	73.567 (25.6859)	72.000 (60.350–86.000)	88.350 (37.2645)	88.350 (75.175–101.525)	83.160 (27.1657)	76.150 (65.750–94.300)
E/A	1.090(0.3818)	1.003 (0.843–1.100)	1.091 (0.2774)	1.100 (0.864–1.252)	1.013 (0.0999)	1.036 (0.970–1.068)	0.764 (0.1003)	0.738 (0.708–0.806)	1.055 (0.3231)	1.000 (0.841–1.158)

*AO, aortic root dimension at end-diastole diameter; BW, Body weight; E/A, ratio of peak velocity blood flow from left ventricular relaxation in early diastole (E wave) to peak velocity flow in late diastole caused by atrial contraction (A wave); Fs, fractional shortening; IVSd, interventricular septal thickness in diastole; IVSs, interventricular septal thickness in systole; LA, left atrial; LVIDd, left ventricular end diastolic internal diameter; LVIDs, left ventricular end systolic internal diameter; LVPWd, left ventricular posterior wall end diastole; LVWs, left ventricular free wall thickness in systole; VHS, vertebral heart size*.

* *As the numbers of dogs under the category “other small breeds” were very small, we did not calculate the IQR for this category; however, 10 dogs of this category were included in the calculation of the total number of dogs*.

** *LVIDDN calculated as per the scaling exponent value of 0.294*.

The PIs (2.5, 5, 25, 50, 75, 95, and 97.5%) for the constant term independent of the BW of the constructed model were calculated and summarized in Table 2. If an M-mode measurement is divided by BW raised to the power shown in the exponent column of Table 2, the result is the normalized or indexed value. This value can be compared with the constants in the table to determine whether they are within the normal range.

**Table 2:** List of constant terms for indexing the M-mode measurements and scaling exponents from the allometric models for calculating prediction intervals of the toy breed dogs (*n* = 86).

**Measurement**	**97.5 Percentile**	**95 Percentile**	**75 Percentile**	**50 Percentile**	**25 Percentile**	**5 Percentile**	**2.5 Percentile**	**Exponent**
LVIDd	1.57	1.52	1.36	1.26	1.17	1.05	1.02	0.332
LVIDS	1.15	1.08	0.89	0.78	0.69	0.57	0.54	0.263
LVPWd	0.57	0.54	0.47	0.43	0.39	0.35	0.33	0.275
LVWS	0.75	0.72	0.62	0.57	0.51	0.45	0.43	0.310
IVSd	0.77	0.67	0.45	0.34	0.26	0.18	0.15	0.486
IVSS	0.83	0.79	0.69	0.62	0.56	0.49	0.46	0.238
AO	1.18	1.13	1.00	0.91	0.83	0.73	0.70	0.183
LA	1.30	1.22	1.03	0.92	0.82	0.69	0.65	0.345

*AO, aortic root dimension at end-diastole diameter; IVSd, interventricular septal thickness in diastole; IVSs, interventricular septal thickness in systole; LA, left atrial; LVIDd, Left ventricular end diastolic internal diameter; LVIDs, left ventricular end systolic internal diameter; LVPWd, left ventricular posterior wall end diastole; LVWs, left ventricular free wall thickness in systole*.

The 95% PIs that appear in Figure and Table 3 were calculated using Equation 1.


(1) $$\begin{array}{r} Yc\pm tSx,y\sqrt{1+\frac{1}{n}+\frac{{\left(x-X\right)}^{2}}{\sum {\left(x_{i}-X\right)}^{2}}} \end{array}$$

In the above equation, Yc is the predicted value of Y for a given value of x, n is the number of data points, t is the Student's t value for n-2 degrees of freedom, Sx,y is the standard error of the estimate, X is the mean of the individual x values, and S (xi-X)^2^ is the sum of the squared deviations of the sample mean.

**Table 3:** Mean value and 95% prediction intervals of the toy breed dogs (*n* = 86).

**Body weight (kg)**	**95% PI**							
	**LVIDd (cm)**	**LVIDS**	**LVPWd (cm)**	**LVWS**	**IVSd (cm)**	**IVSS**	**AO (cm)**	**LA (cm)**
1.5	1.4 (1.2–1.8)	0.9 (0.6–1.3)	0.5 (0.4–0.6)	0.6 (0.5–0.8)	0.4 (0.2–0.9)	0.7 (0.5–0.9)	1.0 (0.8–1.3)	1.1 (0.8–1.5)
2	1.6 (1.3–2.0)	0.9 (0.7–1.3)	0.5 (0.4–0.7)	0.7 (0.5–0.9)	0.5 (0.2–1.0)	0.7 (0.6–1.0)	1.0 (0.8–1.3)	1.2 (0.8–1.6)
2.5	1.7 (1.4–2.1)	1.0 (0.7–1.4)	0.6 (0.4–0.7)	0.8 (0.6–1.0)	0.5 (0.3–1.1)	0.8 (0.6–1.0)	1.1 (0.8–1.4)	1.3 (0.9–1.7)
3	1.8 (1.5–2.2)	1.0 (0.7–1.5)	0.6 (0.5–0.8)	0.8 (0.6–1.0)	0.6 (0.3–1.2)	0.8 (0.6–1.1)	1.1 (0.9–1.4)	1.3 (1.0–1.8)
3.5	1.9 (1.6–2.3)	1.1 (0.8–1.6)	0.6 (0.5–0.8)	0.8 (0.6–1.1)	0.6 (0.3–1.3)	0.8 (0.6–1.1)	1.1 (0.9–1.5)	1.4 (1.0–1.9)
4	2.0 (1.6–2.5)	1.1 (0.8–1.6)	0.6 (0.5–0.8)	0.9 (0.7–1.1)	0.7 (0.3–1.4)	0.9 (0.7–1.1)	1.2 (0.9–1.5)	1.5 (1.1–2.0)
4.5	2.1 (1.7–2.6)	1.2 (0.8–1.7)	0.7 (0.5–0.8)	0.9 (0.7–1.2)	0.7 (0.3–1.5)	0.9 (0.7–1.2)	1.2 (0.9–1.5)	1.5 (1.1–2.1)
5	2.2 (1.8–2.7)	1.2 (0.8–1.7)	0.7 (0.5–0.9)	0.9 (0.7–1.2)	0.8 (0.3–1.6)	0.9 (0.7–1.2)	1.2 (1.0–1.6)	1.6 (1.2–2.2)

*AO, aortic root dimension at end-diastole diameter; IVSd, interventricular septal thickness in diastole; IVSs, interventricular septal thickness in systole; LA, left atrial; LVIDd, Left ventricular end diastolic internal diameter; LVIDs, left ventricular end systolic internal diameter; LVPWd, left ventricular posterior wall end diastole; LVWs, left ventricular free wall thickness in systole*.

The predicted values and 95% PIs for the cases of 1.5, 2.0, 2.5, 3.0, 3.5, 4.0, 4.5, and 5.0 kg of BW of the constructed model were calculated and summarized (Table 3).

## Result

A total of 86 client-owned dogs with a BW of <5 kg were assessed. Among these dogs, there were 40 Toy Poodles, 25 Chihuahuas, seven Yorkshire Terriers, four mongrels, and 10 dogs of other small breeds (two miniature dachshunds, two Maltese, three Papillons, one Shih Tzu, and two mixed breeds). Descriptive data on the basic statistics of the dogs are shown in the Table 1. The mean [± standard deviation (SD)] and median age of these dogs were 6.186 (±3.5097) and 6 years [Inter quartile range (IQR): 25–75%, 3–9 years], respectively. The mean (±SD) and median BW of these dogs were 2.869 (±0.7985) and 2.780 kg (IQR; 25–75%, 2.200–3.300 kg), respectively. As the numbers of dogs under the category “other” were very small, we did not calculate the IQR for this category; however, ten dogs of this category were included in the calculation of the total number of dogs.

Figure shows left ventricular end-diastolic diameter (LVIDd) vs. BW (kg), showing the regression line and the 95% prediction interval for this variable. Since the distribution is curvilinear, it needs to be standardized in groups below 5 kg (8). We standardized this graph by drawing a parallel median so that the standardization equation is considered true even for the group below 5 kg.

Table 2 lists the constants for the indexation of the echocardiographic measurements. These constants that can be employed to recalculate the PIs shown in the plots or to calculate the range of reference values for dogs of any weight were deduced as previously (8). Scaling exponent b for LVIDd was calculated as 0.332. Table 3 shows the mean values and 95% PIs as the normal range for various M-mode measurements for various variables for dogs of different weights (1.5–5.0 kg).

## Discussion

The results of our study highlighted that the linear M-mode dimensions for smaller breed dogs are normalized by dividing the measurement by BW raised to a power in the range of 0.24–0.54. BW is commonly used as a substitute for body size and is often used in statistical regression models featuring a possible non-linear relationship between BW and echocardiographic variables. Using such regression models, reference ranges can be calculated as the PIs for specific BW values (17). In our study, the LVIDDN value for 95% of dogs weighing <5 kg was achieved by dividing the M-mode measurement by BW raised to the power 0.332. This value is different from the exponent value 0.294 included in the calculation of the existing reference threshold value of LVIDDN to identify stage B MMVD (8).

As our study included dogs weighing <5 kg, while calculating constants for the allometric formulas and PIs, the effect of BW on measurements of LVIDD was found to be different compared to that described previously. For example, in the study by Cornell et al. the constants for the variable LVIDD at 2.5 and 97.5 percentile were 1.27 and 1.85, respectively, while in our study, these values were 1.02 and 1.57 (8). The impact of including only small breed dogs in the study was reflected in the difference in relevant LVIDDN values. According to the study by Cornell et al. for a dog of 4.0 kg, the reference range for the variable LVIDD is 1.27 × 4.00.294 = 1.90 cm to 1.85 × 4.00.294 = 2.76 cm (8). However, according to our analysis, if the scaling exponent (constant b) of 0.332 is used, the reference range for the variable LVIDD is 1.02 × 4.00.332 = 1.61 cm to 1.57 × 4.00.332 = 2.48 cm.

Dog breeds are known to affect the incidence of heart disease. For nearly 75% of breeds with an average BW <9 kg, cardiovascular issues were found to be a major cause of death compared to 25% of breeds with average weight >9 kg (5). Moreover, as the chance of MMVD increases with age, the chance of the disease is higher in small dogs, as they have a relatively extended lifespan (18, 19). The incidence of differences in some echocardiographic parameters and values between various dog breeds is related to the wide dissimilarity in the body size and weight, and conformation, as well as physical activities among dogs, limit the clinical usefulness of these parameters (15, 20–22). Addressing the requirements of breed-specific reference values for physiological parameters, several studies have evaluated the reference ranges of M-mode echocardiographic measurements for the normal dog population of various breeds (10, 11, 17). In an earlier study with Miniature Poodles, the reported LVIDDN value was 1.6 ± 0.4 (23). In our study, the LVIDDN value for Toy Poodles was 1.342 ± 0.1164. This difference can be attributed to the size difference between Miniature Poodles and Toy Poodles (5–10 kg vs. <5.0 kg) (24).

In this study, echocardiographic and radiographic evidence were considered to identify the clinical stage of the disease. For example, in the “EPIC study,” the three heart size criteria to indicate for cardiomegaly and start pimobendane treatment were LA/Ao ≥1.6, LVIDDN ≥1.7, and vertebral heart sum (VHS) >10.5—which means even if the other criteria are met, treatment would not start unless the LVIDDN is ≥1.7, the reference threshold comparable to the around 90.0 percentiles of the population in the study by Cornell et al. (8). While the EPIC study concluded that the observation could be extrapolated to the dogs of any bodyweight, it highlighted the need for further evidence.

Taking all 86 dogs into account, when we calculated LVIDDN as measured LVIDD/BW0.294, according to the ACVIM consensus statement, the median LVIDDN value was 1.3215 and the mean value was 1.3204, the observed minimum and maximum were 0.9214 and 1.6072, respectively, while in the first and third quartiles, the values were 1.2472 and 1.4140, respectively (Table 1). Notably, none of these values approached 1.7, the threshold value proposed previously, and was clinically followed by the ACVIM consensus statement (Supplementary Figure 1 available in Supplementary Material on-line). A natural corollary of this observation is that since the normal range for dogs <5 kg is <1.7, the possibility is high that by the time these toy breed dogs start receiving treatment, according to the current guidelines of diagnosis criteria of stage B2 of MMVD, they already reach a very advanced stage of the disease. Previous reports, including the EPIC study, focused mainly on dogs with BW >4 kg, and the diagnostic criteria for toy breed dogs remain mostly unexplored. Therefore, along with diagnostic criteria, we should consider starting treatment as B2 for dogs weighing <5 kg with an LVIDDN of ≥1.6.

Our study is one of the first studies to understand the breed-specific aspects of MMVD in Toy Poodles and Chihuahuas; however, there are a few limitations. Data were collected retrospectively. Being a single-center study with the same operator performing all echocardiographic examinations, no interobserver or intercenter variations were available, which might affect the robustness of the data required to develop new reference values. Furthermore, since we limited the weight to <5 kg, the number of breeds included in the study was limited. However, this has also allowed us to eliminate various confounding factors associated with larger breeds. Further prospective multicenter studies with larger numbers of breeds are warranted to strengthen the clinical implications of our results.

## Conclusion

Body weight-dependent normalized LVIDd references were generated for small breed dogs weighing <5 kg, which are high-risk breeds for MMVD. As one of the first studies on reference echocardiographic parameters relevant for classifying the disease stage, our study is likely to impact future clinical practice in small animal cardiology. Instead of the current threshold of LVIDDN ≥1.7, we propose a value of ≥1.6 to be included in the B2 criterion of the ACVIM consensus statement and guide clinicians in deciding when to start treatment of mitral valve disease in small breed dogs for small breed dogs.

## Data Availability Statement

The original contributions presented in the study are included in the article/Supplementary Material, further inquiries can be directed to the corresponding author.

## Ethics Statement

The study protocol was reviewed and approved by the Institutional Review Board of Ueno no Mori Animal Hospital (approval number 1404-01).

## Author Contributions

NI, YU, and KS contributed to conception and design of the study. KS, EM, MW, and TT organized the database. NI performed the statistical analysis and wrote the first draft of the manuscript. All authors contributed to manuscript revision, read, and approved the submitted version.

## Conflict of Interest

The authors declare that the research was conducted in the absence of any commercial or financial relationships that could be construed as a potential conflict of interest.

## Publisher's Note

All claims expressed in this article are solely those of the authors and do not necessarily represent those of their affiliated organizations, or those of the publisher, the editors and the reviewers. Any product that may be evaluated in this article, or claim that may be made by its manufacturer, is not guaranteed or endorsed by the publisher.

## Supplementary Material

Supplementary Figure 1 Comparison of calculations used in ACVIM recommendation and our study. **(A)** The prediction interval is calculated according to the result of the study by Cornell et al. and our study is represented by the 50% marker X and the 95% upper and lower limit bars. **(B)** In the box-and-whisker diagram, the quartile range is represented by the result of calculating the median using the scaling component b = 0.294, the value used in the ACVIM recommendation.
