# Supplementary figures and images for: Reference Values of M-mode Echocardiographic Parameter in Adult Toy Breed Dogs
Source: Front Vet Sci. 2022 Jun 23;9:918457. doi: 10.3389/fvets.2022.918457 (PMC9261867; doi:10.3389/fvets.2022.918457)

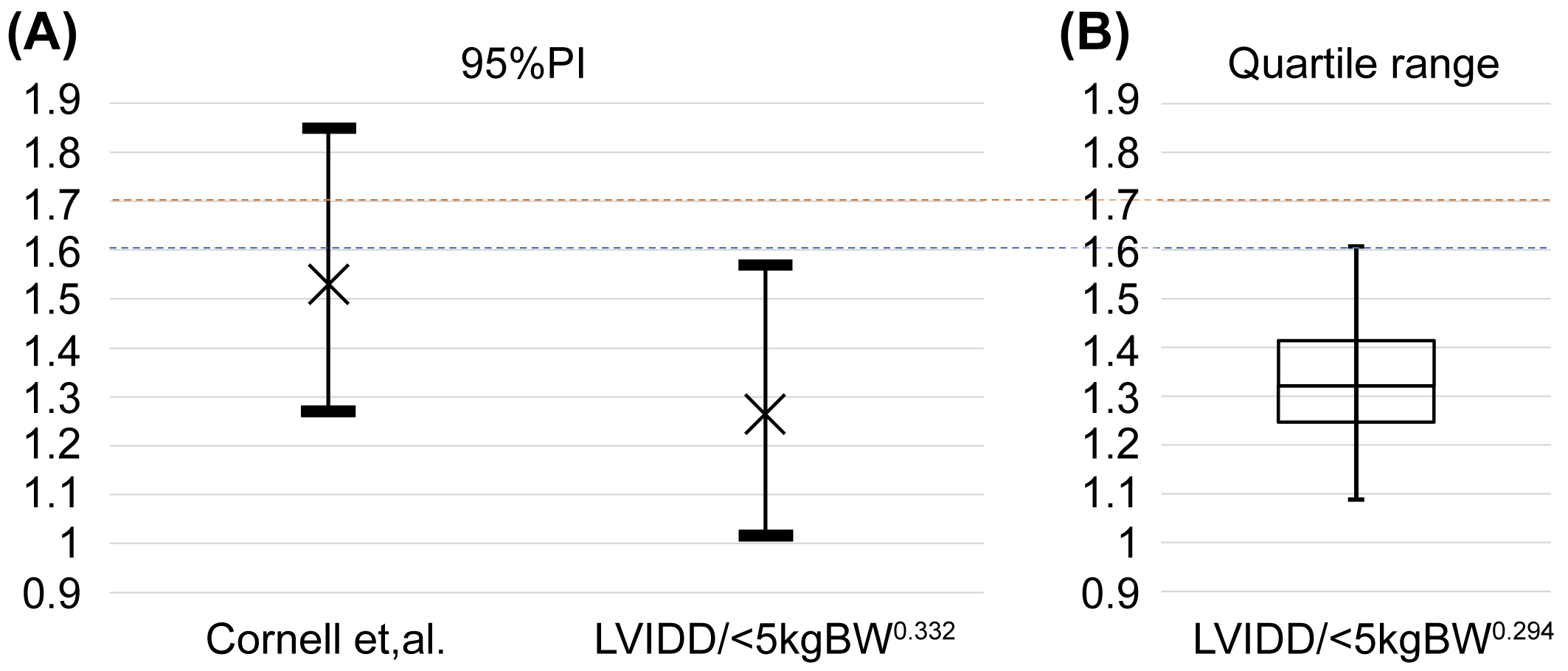

Supplement: Supplementary Figure 1 — Comparison of calculations used in ACVIM recommendation and our study. (A) The prediction interval is calculated according to the result of the study by Cornell et al. and our study is represented by the 50% marker X and the 95% upper and lower limit bars. (B) In the box-and-whisker diagram, the quartile range is represented by the result of calculating the median using the scaling component b = 0.294, the value used in the ACVIM recommendation. [file Image_1.TIF]
